# A focused ethnography of the culture of inclusive caring practice in the intensive care unit

**DOI:** 10.1002/nop2.1009

**Published:** 2021-07-28

**Authors:** Hanan Subhi Al‐Shamaly

**Affiliations:** ^1^ Brisbane Qld Australia

**Keywords:** colleagues, environment, family, focused ethnography, health professionals, intensive/critical care, nurses/midwifes/nursing, oneself, organization, patient

## Abstract

**Aim:**

To explore and understand the culture of nurses' multidimensional “caring‐for” practice in intensive care unit (ICU).

**Design:**

A focused ethnography.

**Methods:**

Data were collected from 35 Registered Nurses through participant observations, field notes, documentation reviews, interviews, informal conversations and Participants' additional information forms over 6 months in one ICU. Thematic data analysis was used.

**Findings:**

Different dimensions of nursing caring in ICU were found. The inclusivity of a culture of nurses' “caring‐for” involved the following: oneself, patients and their families, different colleagues, and caring as ecological consciousness in the ICU environment and organization.

## 1 INTRODUCTION

The concept “caring” is complex. Numerous theoretical and operational perspectives of caring within the context of nursing have emerged. The ongoing discussion about what constitutes caring within nursing practice domains is nowhere more evident than in the adult ICU specialization, where humanistic caring is juxtaposed with advanced technology. Caring in ICU has been discussed in terms of one entity as the patient (Cutler et al., 2013), their family (Mackie et al., 2017), nurses (Hales et al., 2018), health professionals (Handberg & Voss, 2018) or a couple of entities such as patients and nurses (Happ et al., 2014). However, ICU is not limited to caring for patients, their families and nursing staff. This study examines the multidimensional inclusive nature of caring in ICU.

## 2 BACKGROUND

Several authors discussed different caring ICU entities from various perspectives, including patients' perspectives and experiences of care (Cutler et al., 2013), patients' descriptions of the art of nursing (Gramling, 2004), and patients' perceptions of nursing care quality as evidenced by nurse caring behaviours (Reiss, 2005). Other studies examined family members' satisfaction with critical care (Karlsson et al., 2011), family needs (Buckley & Andrews, 2011), family presence (Charlton, 2015) and family involvement in ICU (Mackie et al., 2017; Mitchell et al., 2017). Visiting hours and balance among the needs of patients, families and staff have also been explored (Nyholm & Koskinen, 2017).

Studies on nurses' experiences in critical care settings focused on critically ill patients (Butt, 2010), older adults (Winters, 2012), obstetric (Engström, 2014), bariatric patients (Robstad et al., 2018) and nurses' management of patients' health conditions in ICU (Welch & Barksby, 2011). Researchers investigated sensitive topics, including critical care nurses' perceptions of end‐of‐life (EOL) care (Taylor et al., 2008), postmortem care (O'Connor, 2016), perceptions and responses to ethical and moral distress decisions (Shorideh et al., 2012), and withdrawal of ICU treatment (Templeman, 2015).

Studies on nurses as a workforce and the ICU covered nurses' psychological stress, burnout, grief and debriefing experiences (Akinwolere, 2016), nursing practices (El‐Soussi & Asfour, 2017; Milhomme et al., 2018), professional autonomy and job satisfaction (Ntantana et al., 2017), management and challenges (Ogle & Glass, 2014). Also included were communication in critical care settings (Handberg & Voss, 2018), nurse–doctor communication and interactions (Manias & Street, 2001a, 2001b), interprofessional liaison (Kendall‐Gallagher et al., 2017), nurse–patient communication (Happ et al., 2014), families (Adams et al., 2017) and work hours (Aveyard, 2016).

The wider question of the conjunction of humanistic and technological aspects of caring, which characterizes ICU, is rarely addressed (Alasad, 2002; Nascimentol & Erdmann, 2009). Generally, the nature of caring in ICU discussed only one or two entities or topics. However, caring in ICU involves more than just patients, families and staff. This study looks at the wider range of caring in an ICU. The purpose of this study is to explore and understand the culture of nurses' multidimensional caring in ICU.

## 3 THE STUDY

### 3.1 Design

A focused ethnography, widely acknowledged as a research method for studying behaviours and social interactions in health care, was used (Roberts, 2009). Focused ethnography has useful applications in primary care and hospital healthcare practices and is frequently used to determine ways to enhance care and care processes (Cruz & Higginbottom, 2013). The study was undertaken by the author (Ms. H.A.), a Registered Nurse (RN) with long nursing experience in different fields (intensive care, perioperative and emergency). The Consolidated Criteria for Reporting Qualitative research (COREQ) Checklist was employed to report the study (File S1).

### 3.2 Setting

The study was conducted at the adult ICU of a large private hospital in the Queensland metropolitan area, Australia.

### 3.3 Sample

The ICU Clinical Nurse Manager (CNM) distributed recruitment letters to nursing staff and placed flyers around the nurses' stations and the staff tearoom. Letters and flyers outlined the study purpose, data collection process and Participant's time commitment. Then, the CNM introduced the researcher to the staff, with whom she had had no prior relationship. Each participant (P) received an information sheet, informed consent form and demographic questionnaire from the researcher.

Purposive sample was selected, and the inclusion criteria for participation were as follows: RNs, either gender; employed full‐time; a minimum of 1 year experience in the unit; working rotating shifts; and willing to be interviewed and observed within the practice setting. Thirty‐eight RNs consented to participate but three withdrew because of family issues and heavy workload commitment, resulting in 35 Ps, and Table 1 provides Ps' demographics.

**TABLE 1:** Basic demographic data sheet for study participants

Age	Gender	Marital status	Ethnic background	Languages	Religion	Education	Years' experience in ICU
22–60	F: 29	Married	Australian	English	Catholic	Masters	Range 1–34
	M: 6	25	25	Indian	12	6	1 year = 1
		Partner	New Zealander	Mandarin	Protestant	Bachelors	3 years = 1
		1	1	Chinese	3	17	4 years = 1
		Engaged	British	Tagalog	Anglican	Diploma	5 years = 1
		1	4	Malayalam	1	2	6 years = 4
		Divorced	Irish		Church of England	Postgraduate certificate	7 years = 1
		1	1				8 years = 4
		De facto	Indian		1	7	9 years = 1
		3	1		Buddhist	Graduate certificate for critical care	10 years = 3
		Single	Filipino		2		12 years = 2
		4	1		Hindu		13 years = 1
			Thai		2	3	14 years = 1
			1		Pentecostal		15 years = 3
			Chinese		1		16 years = 1
			1		Honours & respects all religions		19 years = 1
							22 years = 1
							25 years = 1
					1		28 years = 1
					No religious affiliation		30 years = 2
							32 years = 1
					12		34 years = 3
Total participants	35						

### 3.4 Data collection

Data were triangulated from different sources: P's observation, document reviews and interviews, and further written information from Ps was collected.

*Unstructured observation* was used to obtain detailed descriptions of behaviours as they occurred or shortly afterwards by completing a reflective journal or field notes (Borbasi & Jackson, 2012). The observations included the physical ICU layout, characteristics of Ps, activities and social interactions, frequency, and duration of events, precipitating factors, organization, and incidents. Ps were observed closely for two shifts; with some being observed for more shifts because they interacted with other Ps and events.

*Document reviews* coincided with the P observation period. Documentation included patients' files, nurses' notes, charts, communication book, policies and procedures. Reviews focused on obtaining greater insight into nurses' responses to patients and their relatives. This allowed access to data was difficult to acquire through direct observation, interviewing and questioning (Holloway & Wheeler, 2010).

Pilot *interviews* with four Ps allowed me to pre‐test and improve the interview guide and process before full implementation. The interviews were audio‐recorded and arranged to suit the Ps' schedules. Interviews were conducted at workplace (e.g. conference room) and Ps' homes). Interviews started with general questions to encourage Ps to discuss their experiences “What do you believe constitutes caring in ICU?” or “How would you describe caring practices/behaviours/attitudes in ICU?”. Probes and prompts, such as “tell me more about that,” were used to clarify content and augment the information provided. The interviews lasted 1–1.5 hr. I took notes before, during and after the interviews. There were 44 follow‐up interviews to obtain further clarifications from observations.

After the interviews, I asked the Ps to complete a P's additional written information form (PAWIF) if they wished to add further information. PAWIFs were only collected on completion of the data gathering process.

The researcher did not return the interview transcripts to participants for comments, as she conducted *instant member checking* through employing various strategies such as seeking clarification by probing, paraphrasing, using open‐ended questions and listening with an interpretive intent during interviews. Further, member checking was not used (McConnell‐Henry et al., 2011). After 6 months of the fieldwork, no new data were forthcoming, and the study had reached data saturation (Polit & Beck, 2017).

### 3.5 Data analysis

Assisted by NVivo 11^®^ data management software, I analysed the data inductively and thematically in six phases: data immersion, coding, categorizing/sub‐theming, theming, conceptual model development and reporting. Data were segmented, compared, contrasted, synthesized, categorized and conceptualized to identify common codes, subthemes and core themes, from which a mental map of the findings was constructed to capture the core concepts in the data set (Hennink et al., 2011).

### 3.6 Trustworthiness

Ensuring trustworthiness includes credibility, dependability, confirmability, transferability, authenticity and reflexibility (Lincoln et al., 2011; Polit & Beck, 2017). *Credibility* was maintained by the prolonged engagement in the field and data triangulation (Polit & Beck, 2017). *Dependability* was achieved through consistency in the methods of data collection and analysis and triangulation. *Confirmability* was achieved by checking of the codes and analysis to ensure data accuracy, relevance or meaning (Polit & Beck, 2008). *Transferability* included a clear outline the study's context and the rationale for its undertaking, establishing the Ps inclusion criteria and articulating the analysed data. *Authenticity* was assured through obtaining dense and vivid descriptions beyond the researcher's reflexive journal and accurately analyse and represent them and involved ongoing consideration concerning context writing (Polit & Beck, 2017). *Reflexivity* is a critical self‐reflection was assured through the use of reflective journaling about preconceived biases, preferences, and preconceptions that the researcher may have that could influence a situation or interpretation of data (Borbasi et al., 2005; Dowling, 2008; Polit & Beck, 2017).

#### 3.6.1 Reflexivity and the researcher's position

The researcher used reflexivity as an important tool that functions as a reminder that the researcher is an important part of the social world being studied (Hammersley & Atkinson, 2007). The researcher used the constant critical process of a researcher's self‐reflection on personal biases, preferences, values and preconceptions that could affect the processes of data collection and analysis (Doyle, 2013; Polit & Beck, 2017). To preserve reflexivity, the researcher positioned herself through the research process by questioning herself continuously: how have I affected the process of the research, how has the research affected me and where am I now? As recommended by Mulhall et al. (1999). The researcher recorded her reflections in her field notes relating to different phases of data collection. These notes were important as they allowed the researcher to question her influence throughout data collection and analysis, management and final writing. For example, the researcher maintained reflexive notes of her assumptions and behaviours that might have influenced the interviews (Dowling, 2008). The researcher used reflexive practices and journaling as a tool to increase self‐awareness and to monitor interactions between herself and the study participants (Smith‐Sullivan, 2008). Reflexivity also assisted her in examining the contextual factors that constrained the relationships between herself and the participants (Finlay, 2002). As a part of reflexivity, the researcher consciously identified her role as a researcher collecting data from the unit. Therefore, reflexivity allowed her to be aware of her own personal characteristics, previous work experiences, age, gender and education, which had the potential to influence her relationships with the participants. Also, the researcher used a reflective journal to document experiences, fears, problems and general activities surrounding events in the field (Spradley, 1980). As well, the researcher wrote about her experiences of what happened in the unit and reflected on data collection and analysis methods. The researcher undertook reflective journaling before, during and after conducting data collection and analysis. The resulting journals created an account and an audit trail that outlined the progress of the research process (Dowling, 2006; Scott‐Findlay and Estabrooks (2006)). This assisted the credibility of this study and reduced the potential for bias (Polit & Beck, 2017).

During the research, it was important that participants' experiences took precedence over the researcher's own expectations (Roberts, 2007). Therefore, the researcher focused on aspects of the interactions that participants appeared to find hard to articulate. The researcher was mindful of how she presented herself, aware of social positioning within long‐term care. For example, there was a situation where one of the participants communicated with a dying patient even though she was gasping for air until the last moment of her life. The participant then communicated the news of his wife's death to the patient's husband. The researcher discussed the participant's verbal and non‐verbal communication (with both the dead patient and her husband) with the participant and also relayed her own interpretation to get feedback.

Bias is described as predisposition or partiality which can compromise any stage of the research process and produce a distortion of the findings (Polit & Beck, 2017). The researcher was mindful about the possible biases that could affect this study, and these were subsequently addressed from either the participant's or the researcher's side.

To addressing bias from the participants' side, the researcher was aware of the effect of her presence on the participants and the influence on the data (Hammersley & Atkinson, 2007). Therefore, the researcher was careful to minimize her influence on the participants. Participants' lack of candour, or what is called the Hawthorn effect, was originally noted as a possible source of bias (Curry et al., 2009; Speziale, 2007). This occurs when the participant alters their normal behaviour due to their awareness of the researcher's presence or of the situation being scrutinized (Curry et al., 2009; Speziale, 2007). To avoid the “observer effect” and reduce the Hawthorn effect, the researcher's role was non‐intrusive for the participants (Holloway & Wheeler, 2010). This was achieved by the prolonged period of engagement in the unit and with participants. The more time the researcher spent in the unit, the more participants became used to her presence. At times, the researcher used the strategy of covert data collection (concealment) by observing while pretending to be engaged in other activities such as reviewing nurses' notes and charts or by observing participants from the nurses' station.

In addressing bias from the researcher's side, the researcher generally acknowledged her own subjectivity throughout the research process (Ogden, 2008). The researcher managed her bias by being conscious of her values and assumptions and suspending her internal (e.g. beliefs) and external (e.g. environmental factors) presuppositions, biases and experiences to describe the essence of caring in this ICU (Firmin, 2008). In addition, the researcher sought negative case data (Brodsky, 2008; Saumure & Given, 2008), which offered different interpretations of the data. This was experienced in different scenarios, as follows:

The first scenario occurred when participant P29 was allocated to a patient who was a former nurse. This patient was admitted to ICU following a drug overdose because of her addiction. The researcher observed participant P29 treating the patient as an inferior and relaying the patient's story to her colleagues in an unprofessional manner. As both a researcher and a nurse, the researcher felt so upset about the participant's mannerism. She needed to control herself at that point and chose to ask the participant about her attitude in the interview. When asked about this incident, the participant's explanation was accusatory of the patient being addicted. The researcher found it difficult to not respond, as she wanted to focus on listening to the participant's side of the story [P29: field notebook 1].

The second scenario occurred during a bronchoscopy procedure in ICU. During this procedure, the surgeon and participant P11 discovered that a tiny piece of the bronchoscope was missing, and they needed to get another bronchoscope. As an observer with experience in the operating theatre, the researcher suggested use of a three‐way stopcock connection to address this problem. The surgeon and the nursing team appreciated this idea at that time and obtained the three‐way stopcock, which rectified the problem. Unfortunately, it was later discovered that participant P11 complained about this intervention to the unit manager. This incident made the researcher very careful about her participation, even when it was useful. She reminded herself to remain in her role just as an observer and informed the unit manager that she would not interfere in the future. This incident affected the researcher for several days, and she reflected in the field notes to continuously remind herself to be cautious in her research role while conducting this study. Given these scenarios and the conflict in relation to her research position, the researcher realized that she needed to develop a greater capability for reflexivity and mindfulness by acknowledging her position and responding appropriately.

### 3.7 Ethical considerations

Ethics approval was granted by the ethics committees of both the university and the hospital. Informed consent from each P was obtained prior to collecting data. Ps voluntarily agreed to participate by signing the informed consent. Ps could stop participation at any time without consequences. P's confidentiality during and after the study was protected by using pseudonyms and safeguarding data. Patients and other personnel who were not the focus of this study were informed of the reason for the observer researcher's presence.

## 4 FINDINGS

**FIGURE 1:**
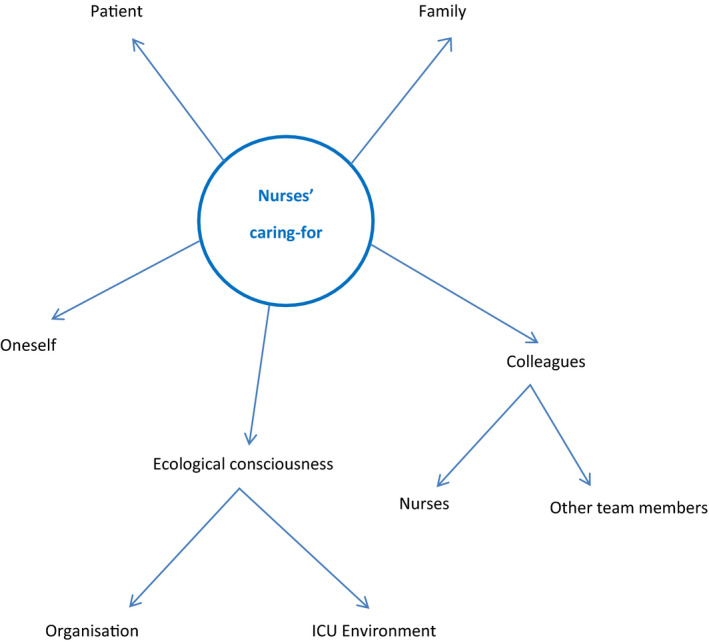
SUN model reflecting the multidimensional culture of “caring‐for” in ICU

To the Ps, the culture of “caring‐for” included many dimensions ingrained as a valued part of ICU's overall caring. Objects of “caring‐for” include oneself, patients, families and colleagues (nurses and other team members). Caring encompasses an ecological consciousness of the ICU environment and wider organization. The SUN model, Figure 1, provides a conceptualization of the various components.

### 4.1 Caring for oneself

Most Ps described caring for oneself activities as a priority within ICU, without which caring for others would be compromised.

Caring and looking after oneself physically, emotionally and psychologically was considered as being professional. Dress and grooming were mentioned as a requirement, for the unit's profile and the positive psychological effect thereof for staff. Observation of staff confirmed the views of P1 who said that outward appearances mattered “because it means I am caring about myself.”

Staff noted the importance of caring for oneself emotionally and psychologically, and in one occasion after patient's death, P36 stated “Having a run of patients dying…can affect you deeply…Seeking professional counselling is viewed by unit staff as a normal process of caring for self.”

P4 captured the general sentiments stating, “you cannot serve from an empty vessel, you need to care for yourself first.”

The general feeling expressed by most Ps are captured by P14 as follows, “Taking breaks…. where having periods of ‘time out’ are viewed by the unit staff as essential for self‐care to provide quality, safe care.”

Nurses viewed work–life balance as an important part of the culture of caring in ICU, P9 articulated:

Staff will take special care to maintain a work‐life balance given the acuity of the unit and the incessant demands placed on staff……When staff seem to be increasingly absorbed by work or are seen to take work home, questions are asked of the person about what is going on?

### 4.2 Caring for patients

Caring for patients is the core business of ICU. The all‐important need for patient safety underpins the need to provide a person‐centred approach. In Ps' PAWIFs, Ps noted their caring for the patients in terms of being “present with”(P36), “aware of the non‐verbal care needs”(P18) and “responsive to the subtle changing health status of patients and their personal illness experience”(P3).

Ps pointed to the primary duty of care to the patients “there is a deep sense of satisfaction knowing that I have dotted every ‘i’ and crossed every ‘t’ and I've gone the extra mile to give my patients the best care possible, and I could not have done any more” (P36).

Almost all Ps agreed that they looked after their patients as they would look after themselves or a family member, “the way nurses look after their patients is the way we look after ourselves, and that is why we always strive to provide the best care possible” (P2).

Several Ps stated they preferred night shifts as it permits providing quality care with fewer distractions, which gives them more time with their patients than during a busy day shift, as P33 said “Night shifts…enables them to spend more time with their patients as they are not distracted by the physiotherapist and visitor… it's nice to have more time and a one‐to‐one relationship with the person you are caring for” (P33).

Tension over the ethics of care often arises between nurses and medical staff concerning maintaining a person on life support and prolonging their life to meet family needs and wishes. The nurses considered prolonging life as an unnecessary trauma to the patient and their family, while the medical staff viewed maintaining a person on life support as sustaining life.

At times I get annoyed at what happens …in terms of keeping people on ventilators. I do not like how doctors push patients to live longer……I think it becomes a bit selfish of the family to let the patient suffer. Ultimately, caring is making sure your patient has the opportunity to have the best outcome in their current situation. (P10)

Ps spoke of the importance of meeting patients' needs at the time of EOL, P1 stated:

We had a patient two weeks ago who was dying. The patient told me she misses her little dog . . . I organised for the husband to have the little dog brought down . . . she saw her dog for the last time …. I think it is part of holistic care to fulfil patients’ needs.

Several Ps shared communication in caring for a dying patient. During observations, I noticed how P19 looked after her dying patient, holding the dying patient's hands and speaking to her softly.

***P19***: “It is ok to go, and hopefully, you are not in pain. Someone is with you; you are not alone.”

***Researcher*** *:* “You are talking to her.”

***P19***: “I found myself privileged to care for dying patients that you spend the last few moments with. Their family members are not there, and therefore, they had somebody with them.”

### 4.3 Caring for families

Ps considered *caring for families* as essential in ICU and described different ways in which they care for their patients' families. P26 stated,

Caring often extends to patient's relatives as well. The time in ICU is highly stressful for them…It is important to demonstrate that you care for them …. keeping them informed, listening to them, and inquiring “what can I do for you?”.

Involving the family in providing care to their loved ones to feel that they have been helpful is important. P12 stated, “we involve the family in the patient's care… if they can do even a tiny little thing, it relieves them.”

During observations, P15 brought a chair to the patient's wife and assisted her in taking a seat. P15 lowered the patient's bed to the same level as the wife's seat. P15 spoke to the wife in a very kind and warm way and asked if she would like a cup of tea while putting her hand on her shoulder [field notebook 2].

Ps P1 and P3 shared their thoughts about caring for relatives, “we need to have very close communication with the family” (P1). “Sometimes families are just so stressed, and all you can do is to provide the information and to be empathic to them” (P3).

Intensive care unit nurses participated in family meetings with health team members for clarification and decision‐making, which was considered essential in providing care to families. P37 said: “usually, we arrange for relatives to talk to the doctor and arrange the time for a family conference.”

I witnessed nurses offer the assistance of the Chaplain or counsellor, informing family about the availability of different supportive resources: “we can always provide them with support from the nurse counsellor if they're really not coping and if they don't have a good network support, or we can bring the Chaplains into the fold, for their spiritual needs” (P3) “We can give the relatives accommodation across the road if they come from out of town. In addition, we can provide free parking tickets for them” (P1).

### 4.4 Caring for colleagues

*Caring for colleagues* comprised two groups: nurse colleagues and other team members. A key aspect of nurses' experiences was support, especially from nurse colleagues and the nurse manager through the atmosphere of assistance and collegiality, as nurses supported each other both physically and emotionally. P29 said:

We look after each other when someone is tired, upset, stressed, or needs a bit of help, we can always go and help them….to make sure they’re not going to get injured, or if they’re busy, you go and see if they want a drink or a cup of tea.

The staff took note of any stresses faced by colleagues experienced outside work that could affect the quality of care. One of the Ps received a phone call from home saying that her husband had suddenly become ill and had gone to the hospital. I observed the nurse manager P1 hugging and assuring P29 as she was crying. The manager enquired how she could help and sent P29 home after ensuring that she was alright [field notebook 2].

Senior nurses provided support to junior nurses who were struggling with the heavy workload of ICU, as experienced by P27 during her early days in ICU:

One of the biggest troubles I have at the moment is prioritising my care to the patient's family….I am still a junior, and I have to learn a lot from the other staff…helping and telling me: ‘you need to tell the family “to leave now”, so you can do your care’.

Agency and casual nursing staff were cared for like the other staff, as P1 stated, “because you don't know their skill level, attitude or time management, you're constantly checking up on them all the time: paperwork, communication, medications, and nursing care to ensure they are safe in their practice”.

An overarching *collegial atmosphere of caring and respect* existed throughout the observations and Ps' descriptions irrespective of the professional discipline. P9 articulated, “we need to look after our co‐workers, whoever they are on our shift—doctors, physiotherapists, cleaners or the people delivering the meals—everybody has a job in ICU. So, we look after our team.”

### 4.5 Caring as ecological consciousness

Caring as ecological consciousness was manifested in two ways: caring for the unit environment and organization.

Ps spoke of the importance of caring for equipment and machines in ICU, and workplace safety and reduction of hazards. P12 stated, “caring in ICU includes caring about the whole unit: caring about the workspace, equipment and that everything is nice, clean and restocked, and it's not in a mess.”

P13 spoke in practical terms about the safety of the workplace:

Environmental‐wise we do the best we can for the space we have in ICU. We need all the equipment; the raised‐up trolleys, chairs, and beds to be located appropriately to have a safe environment to work in. We try to clear the corridors and make things less of a hazard.

P11 echoed the sentiments of the unit concerning repetition in the documentation and how it affects the wider environment:

Everybody says: ‘Why [do] I have to write the same thing three or four times.’ This is just literally wasteful of time, ink, papers, and the storage. The unit is moving towards a paperless unit; we need to move to a paperless society. Too many trees die, and we need to protect the entire environment, our world and universe.

Caring was not limited to ICU environment but extended to the whole organization. P36 spoke about caring for documentation as a part of the organization's requirements:

Well, I suppose it's not so much caring for the patient as caring for the needs of the organisation as well. The organisation's requirement is to do documentation at least once in every 24 hours. So, we're taking care that we document thoroughly.

Most of the Ps, especially in‐charge nurses, articulated their commitment to caring for the organization by emphasizing their concern about the organization's budget for staff and resources. P7 stated, “If we are really busy, we require additional staff to be safe, but at the same time we have to consider the budget.”

## 5 DISCUSSION

The findings from the current study (CS) presented different dimensions of nursing caring in ICU. The inclusivity of a culture of nurses' “caring‐for” involved the following: oneself, patients and their families, different colleagues, and caring as ecological consciousness in the ICU environment and organization.

*For “caring‐for” oneself*. A literature review on maintaining lifestyle balance uncovered two studies (Mealer et al., 2012; Royal College of Nursing, 2005). Mealer et al. (2012) discussed physical health, including sleeping habits, exercise and nutrition. Similarly, the Royal College of Nursing (2005) recommended that nurses maintain a healthy lifestyle to combat the stress of the work environment, including holidays, sufficient rest, a balanced diet, regular breaks during the working day, delegating tasks appropriately, saying “no” as necessary, and not working overtime when they cannot or do not want to. Of particular importance to the Ps in the CS was maintaining a balanced and healthy lifestyle and taking time away from the workplace (e.g. holidays or breaks between shifts).

Intensive care unit nurses identified monitoring their psychological well‐being by accepting opportunities to debrief when necessary, whether through professional counselling, peer support or pastoral care. Also, Ps reported that attending to their spiritual and religious practices helped them maintain a personal sense of well‐being. These findings were compatible with Mealer et al. (2012), who found that nurses seek solace and spiritual energy in difficult times through religious practices such as praying and found that ICU nurses used reiki and pastoral care for the effects of compassion satisfaction and fatigue, and moral distress.

Ps' emotional self‐care included leaving work stress at the workplace and debriefing when required. Similarly, Mealer et al. (2012) found that Ps could separate work from their personal life as a counterbalance to work stress and self‐care. Emotionally distancing oneself from stressful situations, including avoidance and using the psychosocial process of “protecting from stress,” provides the necessary barrier for self‐preservation (Mitchell, 2011; Siffleet et al., 2015).

*“Caring‐for” the patient*. Providing person‐centred care was a central concern for the study Ps—being with the person in their illness and acknowledging the person behind “the patient” and advocating on their behalf. Similar findings were described in other studies (Benner, 2002; Carvalho & Lunardi, 2009; Hasse, 2013; de Lima Guimarães et al., 2017), where providing a patient‐centred approach to care was stressed. Ensuring patient safety, especially for unconscious patients and those on mechanical ventilation, was another area of concern to Ps, confirming findings by Gimenes et al. (2016) and Karlsson and Bergbom (2015).

Ps in the CS encountered difficulty attending to patients' psycho‐emotional and especially spiritual needs at the EOL stage. Studies focusing on EOL experiences for patients and nurses' ability to respond appropriately found nurses pay more attention to patients' physical than psychological and spiritual needs, especially in the EOL stage (Canfield et al., 2016; McCallum & McConigley, 2013; Tyler, 2017). Perceived ethical dilemmas concerning decisions to withhold treatment or prolong life to alleviate family distress while maintaining the quality of life for the patient added to nurses' already complex workload.

Similar findings were identified Campbell (2015) and Carvalho and Lunardi (2009). Amid such ethical dilemmas, Ps discussed their role as advocate in ensuring that the patients' needs are met, preserving the dignity of the person. Similarly, King and Thomas (2013) highlighted the importance of being truthful, advocating, remaining connected with the patient and making the death as comfortable, peaceful and dignified as possible. This includes being with dying patients, managing pain and comfort, attending to wishes, promoting earlier cessation of treatment and not initiating aggressive treatment (Beckstrand et al., 2006), exactly as noted in the CS.

Regarding *“caring‐for” family*, Ps in this study aimed to keep the family well informed and updated about the patient's health. Previous studies also noted the importance of providing and updating families with information about the patient (Attia et al., 2013; Cannon, 2011; Carlson et al., 2015). Also, Ps reported involving the family in their loved one's care included feeding, family conferences/meetings to discuss the person's condition and treatment. Ågård and Maindal (2009) and Davidson (2009) identified the need to involve the family in care and treatment decisions and promote the family's involvement in their loved one's daily care. Further, the need to respect, listen and adhere to the family's views and concerns was evident in previous studies (Cannon, 2011; Carlson et al., 2015; Davidson, 2009; Khalaila, 2014), in which attentive listening by nurses to relatives' needs was instrumental in alleviating the anxiety and distress of families in ICU. Ps in this study found identifying the level of support required by each family to meet their needs and expectations in the care of their loved ones important. Consideration of the relatives' closeness to the patient informed determining the types of support and assistance needed. Åsa and Siv (2007), Blanchard and Alavi (2008), and Buckley and Andrews (2011) identified the closeness of the family members to the patient as vital in the provision ICU care, in addition to providing psychological support to families (Celik et al., 2008; Nordgren & Olsson, 2004). Ps in this study explicated that families rely on open communication, considered as core to building a trusting relationship between families and staff, which is imperative in working with the patient and family and facilitating understanding of the patient's condition and prognosis, especially at the EOL stage. Similar findings were reported in previous studies (Attia et al., 2013; Ranse et al., 2012; Rushton et al., 2007). A further consideration in developing a trusting, supportive relationship with family was in the area of family conflict (Attia et al., 2013; Esmaeili et al., 2014).

Ps' provision of care and support did not end when the patient died; sometimes, it continued beyond the patient's death with follow‐up contact with the family to provide support, send flowers on behalf of the unit, and if appropriate and possible, attend the patient's funeral. Such findings were also identified in other studies, in which staff believed part of their role was to continue providing support and to advocate on behalf of the family if necessary (Bloomer & O’Connor, 2012; Fridh et al., 2007; Virginio et al., 2014).

*“Caring‐for” nurse colleagues*. The consensus within the literature is that unit managers play a pivotal role in providing psychological, emotional and spiritual support for staff who are confronted daily with high levels of work stress (Efstathiou & Walker, 2014; Tirgari et al., 2013; Walker & Deacon, 2016). In this study, nurses mentioned the daily pressures of dealing with life and death situations, having to be present for each other in stressful moments. Consistent with this study, the value of compassionate leadership that advocates on behalf of nurse colleagues was identified by Todaro‐Franceschi (2013).

In the CS, debriefing by staff or professional counselling and pastoral care was available to staff in times of stress and crisis. This aligned with Jordan et al. (2014) principle that immediate debriefing, promoting communication among multidisciplinary team members, and accessing counselling or pastoral care are important considerations in stressful areas such as ICUs. Equally, Beumer (2008) and Halcomb et al. (2004) identified the need for professional workshops and education about grief, loss and coping in situations where loss and grief are ever present. France et al. (2011) suggested that trust, mutual respect, mentoring, collegiality and camaraderie are necessary for creating a healing and supportive environment. Similar findings were yielded in this study, as Ps spoke of and were observed to live the values of support for each other, assist colleagues struggling with workloads, experiencing stressful moments or feeling overwhelmed, and working alongside new staff to ensure appropriate orientation to the unit.

The unit manager and in‐charge nurses paid attention to the allocation of workloads, particularly to staff rostered on 12‐hr shifts, especially when nurses expressed concern about their workloads and patient safety concerns. Subsequently, workload was reviewed by the in‐charge, and resources were allocated to minimize staff concerns. Similar findings were reported by Richardson et al. (2007) who found that nurses could safely work maximally three consecutive 12‐hr day shifts or four consecutive 12‐hr night shifts.

The complexity of patients' care needs acts as additional workload. Patients become agitated based on their health status and a “no sedation” unit protocol. Complex care posed additional challenges, including the need to view patient care as an interdisciplinary responsibility (Laerknera et al., 2015). In such situations, Ps were quick to take over some caring responsibilities to ensure their colleagues were not exposed to unsafe practices.

*“Caring‐for” other health team colleagues*. There was mutual respect between all health providers and different styles of communication for different situations in the CS, especially in critical times. Piquette et al. (2009) investigated ICU healthcare professionals' perceptions, including nurses, physicians, and respiratory therapists, regarding how medical crises affect their team interactions during the three stages: pre‐crisis, during crisis, and postcrisis. The study found that team interaction was based on mutual respect for expertise during the “pre‐crisis” period. However, “during crisis” period, there was a lack of civility. In the “postcrisis” period, divergent perceptions among health professionals and team dispersion left nurses with queries that could be addressed only partially by discussion and feedback. Rose (2011) highlighted the importance of interprofessional communication and collaboration in ICU through shared goals, values and partnerships, including explicit, complementary and interdependent roles, mutual respect and power sharing. These findings are congruent with the CS regarding collaboration, mutual respect and professional communication between different health professionals. Rushton (2006) noted that critical care nurses' (CCN) leaders are instrumental in creating an environment conducive to ethical practice by engaging interdisciplinary colleagues in creating a shared commitment to transform the critical care environment, explore moral distress symptoms and find solutions and coping strategies for moral distress issues. This was evident in the CS, in which the health team members always tried to find solutions and coping strategies during times of moral distress.

*“Caring‐for” ICU environment*. Ps in this study used different ways to create a healthy and safe environment. Issues, including noise, light, colour, temperature and comfort, were ameliorated through creative design, family and pet visitation, and sleep promotion. These findings are comparable with those of Fontaine et al. (2001), suggested designing a humanistic critical care environment and stressed ensuring a safe environment with adequate supplies, equipment, services, technology, and sufficient and competent nursing staff that provide high‐quality care and a healthy work environment and reduce stress for nurses in critical care settings. Huddleston and Gray’s (2016) defined a healthy work environment as involving physical safety for the patient, family, and staff, and psychological safety, which is comparable with the CS findings.

Similarly, Ceronsky (2001) indicated that a collegial, collaborative, healthy work environment results in patient, family and staff satisfaction. A healthy ICU environment not only enables nurses to attain personal satisfaction in their work but also helps them meet organizational goals (Schmalenberg & Kramer, 2007). I witnessed and Ps expressed that all nurses and health team members strove to provide a safe and healthy ICU environment through skilful communication, multidisciplinary team collaboration, appropriate staffing levels, effective decision‐making, and authentic leadership, and maintaining physical and psychological safety for patients, families, and staff. The provision of a healthy environment has been cited as a priority in previous studies (Huddleston & Gray, 2016; Tracy & Ceronsky, 2001; Tremper, 2004), which meets the standards of The American Association of Critical Care Nurses (AACN) (2005) for establishing and sustaining a healthy critical care work environment.

As with other studies, in this study health team members utilized digital technology to ensure healthy, safe treatment and the environment for patient care in ICU. Electronic health records save time, reduce nursing workloads and facilitate nurses' work by increasing access to patient information (Kleinpell et al., 2016; Kossman & Scheidenhelm, 2008; Wikström et al., 2007).

*“Caring‐for” the organization*. Nurses were mindful of wasteful stocking practices and endeavoured to establish and maintain waste management. This finding corroborated Morrow et al. ’s (2013) findings that reducing waste was a concern for ICU nurses.

To my knowledge, this is the first study discusses different dimensions of nursing caring in ICU. The inclusivity of a culture of nurses' “caring‐for” involved the following: oneself, patients and their families, different colleagues, and caring as ecological consciousness in the ICU environment and organization, specifically the latest one (ecological consciousness). Nevertheless, other studies discussed only one to two entities or topics of nursing caring in critical settings.

### 5.1 Limitations

This study's only limitation is that the findings cannot be considered generalizable to the broader population because of the cultural differences in organization and critical care settings. However, this was the expectation of the study design.

## 6 CONCLUSION

- To date, this is the first study to address the inclusivity of a culture of multidimensional “caring‐for” practice by ICU nurses, which is imperative for the provision of quality health care. This culture focuses on the healthcare needs of oneself, patient, family, multidisciplinary team members, ICU environment and organization.
- The study needs to be underpinned and enhanced in health systems and educational institutions, particularly caring for oneself, clinical environments and organizations.
- It is necessary to find consensus and strategies among health professionals regarding ethical considerations to the patient's rights and treatments in critical care settings, especially in the EOL stage.
- The significance of communicating with patients in the EOL stage is not limited to the time before and during patients' death but extends after their death.
- Reconsidering caring for the family as “an extension of caring for the patient” is significant.
- Intensive care unit nurses are encouraged to participate more effectively in family meetings for their patients' treatment and decision‐making through their personality attributes, confidence, experiences and relationships with other health team members.
- Enhancing the health team members' caring sense as *ecological consciousness* of the clinical settings and health institutions and organizations is significant.
- The culture of inclusive nursing caring practices in ICU could have positive and negative effects on nursing staff and the efficiency of working time, quality of care provided and staff reactions. This requires special consideration from clinicians, managers and policymakers.
- Further research is required to investigate the inclusive caring practices within different ICUs, particularly “caring‐for” oneself, ICU environment and the organization.

Under the umbrella of nursing, nurses are caring for various entities. Unfortunately, usually nurses give the priority to care for others and other things first, and eventually they care for themselves. There is a quote says, “you cannot serve from an empty vessel,” however; it is customary for nurses to take care of others when they desperately need to look after themselves first. This could result with nurses' burnout, absenteeism, turnover, quitting from nursing profession and other negative consequences. At the end of this article, the author wishes to emphasize a recommendation of the necessity that nurses need to be compassionate and taking care of themselves first and foremost before caring for others. Nurses should have a balance in their lives. It is unfair to the nurses to oppress themselves, whether it is in professional or personal life . It is enough for nurses to play the candle's role that consume*s* itself in lighting others. Enough is enough!

## CONFLICT OF INTEREST

The author declares no conflict of interest.

## Supporting information

File S1

## Data Availability

The data that support the findings of this study are openly available in [repository name e.g “figshare”] at http://doi.org/[doi], reference number [reference number].

## References

[nop21009-bib-0001] Adams, A. , Mannix, T. , & Harrington, A. (2017). Nurses' communication with families in the intensive care unit—A literature review. Nurse Critical Care, 22(2), 70–80. 10.1111/nicc.12141 25583405

[nop21009-bib-0002] Ågård, A. , & Maindal, H. (2009). Interacting with relatives in intensive care unit. Nurses' perceptions of a challenging task. Nursing in Critical Care, 14(5), 264–272. 10.1111/j.1478-5153.2009.00347.x 19706077

[nop21009-bib-0003] Akinwolere, O. (2016). Psychological stress in critical care nurses. Walden University.

[nop21009-bib-0004] Alasad, J. (2002). Managing technology in the intensive care unit: The nurses' nurses' experience. International Journal of Nursing Studies, 39(4), 407–413. 10.1016/S0020-7489(01)00041-4 11909617

[nop21009-bib-0005] American Association of Critical Care Nurses (AACN) (2005). AACN Standards for establishing and sustaining healthy work environment: A journey to excellence. American Journal of Critical Care, 14(3), 187–198. Retrieved from http://ajcc.aacnjournals.org/content/14/3/187.short 15840893

[nop21009-bib-0007] Åsa, E. , & Siv, S. (2007). Close relatives in intensive care from the perspective of critical care nurses. Journal of Clinical Nursing, 16(9), 1651–1659. 10.1111/j.1365-2702.2005.01520.x| 17459138

[nop21009-bib-0008] Attia, A. , Abd‐Elaziz, W. , & Kandeel, N. (2013). Critical care nurses' perception of barriers and supportive behaviors in end‐of‐life care. American Journal of Hospice and Palliative Medicine®, 30(3), 297–304. 10.1177/1049909112450067 22743231

[nop21009-bib-0009] Aveyard, D. (2016). How do 12‐hour shifts affect ICU nurses? Kai Tiaki: Nursing New Zealand, 22(11), 34–36. 30556970

[nop21009-bib-0010] Beckstrand, R. , Callister, L. , & Kirchhoff, K. (2006). Providing a ‘good death’: Critical care nurses' suggestions for improving end‐of‐life care. American Journal of Critical Care, 15(1), 38–45. 10.4037/ajcc2006.15.1.38 16391313

[nop21009-bib-0011] Benner, P. (2002). Caring for the silent patient. American Journal of Critical Care, 11(5), 480–481. 10.4037/ajcc2002.11.5.480 12233974

[nop21009-bib-0012] Beumer, C. (2008). Innovative solutions: The effect of a workshop on reducing the experience of moral distress in an intensive care unit setting. Dimensions of Critical Care Nursing, 27(6), 263–267. 10.1097/01.DCC.0000338871.77658.03 18953194

[nop21009-bib-0013] Blanchard, D. , & Alavi, C. (2008). Asymmetry in the intensive care unit: Redressing imbalance and meeting the needs of family. Nursing in Critical Care, 13(5), 225–231. 10.1111/j.1478-5153.2008.00292.x 18816308

[nop21009-bib-0014] Bloomer, M. , & O’Connor, M. (2012). Providing end‐of‐life care in the intensive care unit: Issues that impact on nurse professionalism. Singapore Nursing Journal, 39(3), 25–30.

[nop21009-bib-0015] Borbasi, S. , & Jackson, D. (2012). Qualitative research: The whole picture. Elsevier.

[nop21009-bib-0016] Borbasi, S. , Jackson, D. , & Wilkes, L. (2005). Fieldwork in nursing research: Positionality, practicalities and predicaments. Journal of Advanced Nursing, 51(5), 493–501. 10.1111/j.1365-2648.2005.03523.x 16098166

[nop21009-bib-0017] Brodsky, A. (2008). Negative case analysis. In M. Given (Ed.), The SAGE encyclopedia of qualitative research methods (2, pp. 552). SAGE Publications.

[nop21009-bib-0018] Buckley, P. , & Andrews, T. (2011). Intensive care nurses' knowledge of critical care family needs. Intensive and Critical Care Nursing, 27(5), 263–272. 10.1016/j.iccn.2011.07.001 21868224

[nop21009-bib-0019] Butt, A. (2010). Nursing care of the chronically critically ill: An exploratory descriptive study. Victoria University of Wellington. (Master thesis).

[nop21009-bib-0020] Campbell, M. (2015). Caring for dying patients in the intensive care unit managing pain, dyspnea, anxiety, delirium, and death rattle. AACN Advanced Critical Care, 26(2), 110–120. 10.4037/NCI.0000000000000077 25898878

[nop21009-bib-0021] Canfield, C. , Taylor, D. , Nagy, K. , Strauser, C. , VanKerkhove, K. , Wills, S. , Sawicki, P. , & Sorrell, J. (2016). Critical care nurses' perceived need for guidance in addressing spirituality in critically ill patients. American Journal of Critical Care, 25(3), 206–211. 10.4037/ajcc2016276 27134224

[nop21009-bib-0022] Cannon, S. (2011). Family‐centered care in the critical care setting: Is it best practice? Dimensions of Critical Care Nursing, 30(5), 241–245. 10.1097/DCC.0b013e3182276f9a 21841414

[nop21009-bib-0023] Carlson, E. , Spain, D. , Muhtadie, L. , McDade‐Montez, L. , & Macia, K. (2015). Care and caring in the intensive care unit: Family members' distress and perceptions about staff skills, communication, and emotional support. Journal of Critical Care, 30(3), 557–561. 10.1016/j.jcrc.2015.01.012 25682345 PMC4414707

[nop21009-bib-0024] Carvalho, K. , & Lunardi, V. (2009). Therapeutic futility as an ethical issue: Intensive care unit nurses. Revista Latino‐Americana De Enfermagem, 17(3), 308–313. 10.1590/S0104-11692009000300005 19669039

[nop21009-bib-0025] Celik, S. , Ugras, G. , Durdu, S. , Kubas, M. , & Aksoy, G. (2008). Critical care nurses' knowledge about the care of deceased adult patients in an intensive care unit. Australian Journal of Advanced Nursing, 26(1), 53–58.

[nop21009-bib-0026] Charlton, S.‐G.‐M. (2015). Family presence and visitation in critical care: A rapid evidence assessment. University of British Columbia.

[nop21009-bib-0027] Cruz, E. , & Higginbottom, G. (2013). The use of focused ethnography in nursing research. Nurse Researcher, 20(4), 36–43. 10.7748/nr2013.03.20.4.36.e305 23520711

[nop21009-bib-0028] Curry, L. A. , Nembhard, I. M. , & Bradley, E. H. (2009). Qualitative and mixed methods provide unique contributions to outcomes research. Circulation, 119(10), 1442–1452. 10.1161/CIRCULATIONAHA.107.742775 19289649

[nop21009-bib-0029] Cutler, L. , Hayter, M. , & Ryan, T. (2013). A critical review and synthesis of qualitative research on patient experiences of critical illness. Intensive and Critical Care Nursing, 29(3), 147–157. 10.1016/j.iccn.2012.12.00110.1016/j.iccn.2012.12.001 23312486

[nop21009-bib-0030] Davidson, J. (2009). Family‐centered care: Meeting the needs of patients' families and helping families adapt to critical illness. Critical Care Nurse, 29(3), 28–34. 10.4037/ccn2009611 19487778

[nop21009-bib-0031] de Lima Guimarães, G. , Silqueira de Matos, S. , Ferreira Ferraz, A. , Figueiredo Manzo, B. , Sharry, S. , de Souza, F. , & Aparecida, M. (2017). Rediscover of the sympathy in the practice of the nurse in intensive therapy. Journal of Nursing UFPE/Revista De Enfermagem UFPE, 11(2), 491–497. 10.5205/reuol.10263-91568-1-RV.11022017021

[nop21009-bib-0033] Dowling, M. (2006). Approaches to reflexivity in qualitative research. Nurse Researcher, 13(3), 7–21. 10.7748/nr2006.04.13.3.7.c5975 16594366

[nop21009-bib-0034] Dowling, M. (2008). Reflexivity. In L. M. Given (Ed.), The SAGE encyclopedia of qualitative research methods (pp. 748–749). SAGE.

[nop21009-bib-0035] Doyle, S. (2013). Reflexivity and the capacity to think. Qualitative Health Research, 23(2), 248–255. 10.1177/1049732312467854 23258421

[nop21009-bib-0036] Efstathiou, N. , & Walker, W. (2014). Intensive care nurses' experiences of providing end‐of‐life care after treatment withdrawal: A qualitative study. Journal of Clinical Nursing, 23(21–22), 3188–3196. 10.1111/jocn.12565 25453123

[nop21009-bib-0037] El‐Soussi, A. , & Asfour, H. (2017). A return to the basics: Nurses' practices and knowledge about interventional patient hygiene in critical care units. Intensive and Critical Care Nursing, 40, 11–17. 10.1016/j.iccn.2016.10.002 28131655

[nop21009-bib-0038] Engström, Å. (2014). Activity: Nursing mothers in an ICU after complicated childbirth. Paper presented at the OPTIMISE 2014: Optimising childbirth across Europe, An interdisciplinary maternity care conference, Brussels, Belgium.

[nop21009-bib-0039] Esmaeili, M. , Ali Cheraghi, M. , & Salsali, M. (2014). Barriers to patient‐centered care: A thematic analysis study. International Journal of Nursing Knowledge, 25(1), 2–8. 10.1111/2047-3095.12012 24467836

[nop21009-bib-0040] Finlay, L. (2002). ‘Outing’ the researcher: The provenance, process, and practice of reflexivity. Qualitative Health Research, 12(4), 531–545. 10.1177/104973202129120052 11939252

[nop21009-bib-0120] Firmin, M. (2008). Data collection. M. Given The SAGE encyclopedia of qualitative research methods, 1, (190–193). California, USA: SAGE Publications.

[nop21009-bib-0041] Fontaine, D. , Briggs, L. , & Pope‐Smith, B. (2001). Designing humanistic critical care environments. Critical Care Nursing Quarterly, 24(3), 21–34. 10.1097/00002727-200111000-00003 11858555

[nop21009-bib-0042] France, N. , Byers, D. , Kearney, B. , & Myatt, S. (2011). Creating a healing environment: Nurse‐to‐nurse caring in the critical care unit. International Journal for Human Caring, 15(1), 44–48.

[nop21009-bib-0043] Fridh, I. , Forsberg, A. , & Bergbom, I. (2007). End‐of‐life care in intensive care units–family routines and environmental factors. Scandinavian Journal of Caring Sciences, 21(1), 25–31. 10.1111/j.1471-6712.2007.00470.x| 17428211

[nop21009-bib-0044] Gimenes, F. , Torrieri, M. , Gabriel, C. , Rocha, F. , Silva, A. , Shasanmi, R. , & Cassiani, S. (2016). Applying an ecological restoration approach to study patient safety culture in an intensive care unit. Journal of Clinical Nursing, 25(7–8), 1073–1085. 10.1111/jocn.13147 26876047

[nop21009-bib-0045] Gramling, K. (2004). A narrative study of nursing art in critical care. Journal of Holistic Nursing, 22(4), 379–398. 10.1177/0898010104269794 15486156

[nop21009-bib-0046] Halcomb, E. , Daly, J. , Jackson, D. , & Davidson, P. (2004). An insight into Australian nurses' experience of withdrawal/withholding of treatment in the ICU. Intensive and Critical Care Nursing, 20(4), 214–222. 10.1016/j.iccn.2004.05.010 15288875

[nop21009-bib-0047] Hales, C. , Coombs, M. , & de Vries, K. (2018). The challenges in caring for morbidly obese patients in intensive care: A focused ethnographic study. Australian Critical Care, 31(1), 37–41. Retrieved from http://www.sciencedirect.com/science/article/pii/S1036731417301558://doi.org/ 10.1016/j.aucc.2017.02.070 28320611

[nop21009-bib-0048] Hammersley, M. , & Atkinson, P. (2007). Ethnography: Principles in practice (3rd ed). Routledge.

[nop21009-bib-0049] Handberg, C. , & Voss, A. (2018). Implementing augmentative and alternative communication in critical care settings: Perspectives of healthcare professionals. Journal of Clinical Nursing, 27(1–2), 102–114. 10.1111/jocn.13851 28401613

[nop21009-bib-0050] Happ, M. B. , Garrett, K. L. , Tate, J. A. , DiVirgilio, D. , Houze, M. P. , Demirci, J. R. , George, E. , & Sereika, S. M. (2014). Effect of a multi‐level intervention on nurse–patient communication in the intensive care unit: Results of the SPEACS trial. Heart & Lung: The Journal of Acute and Critical Care, 43(2), 89–98. 10.1016/j.hrtlng.2013.11.010 24495519 PMC4053558

[nop21009-bib-0051] Hasse, G. (2013). Patient‐centered care in adult trauma intensive care unit. Journal of Trauma Nursing, 20(3), 163–165. 10.1097/JTN.0b013e3182a172a0 24005121

[nop21009-bib-0052] Hennink, M. , Hutter, I. , & Bailey, A. (2011). Qualitative research methods. SAGE.

[nop21009-bib-0053] Holloway, I. , & Wheeler, S. (2010). Qualitative research in nursing and healthcare (3rd ed). Wiley‐Blackwell, John Wiley & Sons.

[nop21009-bib-0054] Huddleston, P. , & Gray, J. (2016). Describing nurse leaders' and direct care nurses' perceptions of a healthy work environment in acute care settings, part 2. Journal of Nursing Administration, 46(9), 462–467. 10.1097/NNA.0000000000000376 27556655

[nop21009-bib-0055] Jordan, P. , Clifford, I. , & Williams, M. (2014). The experiences of critical care nurses with regard to end‐of‐life issues in the intensive care unit. Africa Journal of Nursing and Midwifery, 16(2), 71–84.

[nop21009-bib-0056] Karlsson, C. , Tisell, A. , Engström, Å. , & Andershed, B. (2011). Family members' members' satisfaction with critical care: A pilot study. Nursing in Critical Care, 16(1), 11–18. 21199550 10.1111/j.1478-5153.2010.00388.x

[nop21009-bib-0057] Karlsson, V. , & Bergbom, I. (2015). ICU professionals' experiences of caring for conscious patients receiving MVT. Western Journal of Nursing Research, 37(3), 360–375. 10.1177/0193945914523143 24558056

[nop21009-bib-0058] Kendall‐Gallagher, D. , Reeves, S. , Alexanian, J. , & Kitto, S. (2017). A nursing perspective of interprofessional work in critical care: Findings from a secondary analysis. Journal of Critical Care, 38, 20–26. 10.1016/j.jcrc.2016.10.007 27835799

[nop21009-bib-0059] Khalaila, R. (2014). Meeting the needs of patients' families in intensive care units. Nursing Standard, 28(43), 37–44. 10.7748/ns.28.43.37.e8333 25159786

[nop21009-bib-0060] King, P. , & Thomas, S. (2013). Phenomenological study of ICU nurses' experiences caring for dying patients. Western Journal of Nursing Research, 35(10), 1292–1308. 10.1177/0193945913492571 23797098

[nop21009-bib-0061] Kleinpell, R. , Barden, C. , Rincon, T. , McCarthy, M. , & Zapatochny Rufo, R. J. (2016). Assessing the impact of telemedicine on nursing care in intensive care units. American Journal of Critical Care, 25(1), e14–e20. 10.4037/ajcc2016808 26724303

[nop21009-bib-0062] Kossman, S. , & Scheidenhelm, S. (2008). Nurses' perceptions of the impact of electronic health records on work and patient outcomes. CIN: Computers, Informatics, Nursing, 26(2), 69–77. 10.1097/01.NCN.0000304775.40531.67 18317257

[nop21009-bib-0063] Laerknera, E. , Egerodc, I. , & Hansen, H. (2015). Nurses' experiences of caring for critically ill, non‐sedated, mechanically ventilated patients in the intensive care unit: A qualitative study. Intensive and Critical Care Nursing, 31(4), 196–204. 10.1016/j.iccn.2015.01.005 25743598

[nop21009-bib-0065] Lincoln, Y. , Lynham, S. , & Guba, E. (2011). Paradigmatic controversies, contradictions, and emerging confluences. In N. K. Denzin , & N. B. Lincoln (Eds.), The SAGE handbook of qualitative research (4th ed., pp. 97–128). SAGE.

[nop21009-bib-0066] Mackie, B. , Marshall, A. , & Mitchell, M. (2017). Acute care nurses' views on family participation and collaboration in fundamental care. Journal of Clinical Nursing, 27(11‐12), 2346–2359. 10.1111/jocn.14185 29171145

[nop21009-bib-0067] Manias, E. , & Street, A. (2001a). Nurse–doctor interactions during critical care ward rounds. Journal of Clinical Nursing, 10(4), 442–450. 10.1046/j.1365-2702.2001.00504.x| 11822491

[nop21009-bib-0068] Manias, E. , & Street, A. (2001b). Nurses and doctors communicating through medication order charts in critical care. Australian Critical Care, 14(1), 17–23. 10.1016/S1036-7314(01)80018-7 11899756

[nop21009-bib-0069] McCallum, A. , & McConigley, R. (2013). Nurses' perceptions of caring for dying patients in an open critical care unit: A descriptive exploratory study. International Journal of Palliative Nursing, 19(1), 25–30. 23354430 10.12968/ijpn.2013.19.1.25

[nop21009-bib-0070] McConnell‐Henry, T. , Chapman, Y. , & Francis, K. (2011). Member checking and Heideggerian phenomenology: A redundant component. Nurse Researcher, 18(2), 28–37. 10.7748/nr2011.01.18.2.28.c8282 21319482

[nop21009-bib-0071] Mealer, M. , Jones, J. , & Moss, M. (2012). A qualitative study of resilience and posttraumatic stress disorder in United States ICU nurses. Intensive Care Medicine, 38(9), 1445–1451. 22618093 10.1007/s00134-012-2600-6

[nop21009-bib-0072] Milhomme, D. , Gagnon, J. , & Lechasseur, K. (2018). The clinical surveillance process as carried out by expert nurses in a critical care context: A theoretical explanation. Intensive and Critical Care Nursing, 44, 24–30. 10.1016/j.iccn.2017.07.010 28869149

[nop21009-bib-0073] Mitchell, A. (2011). Focusing on mind, body, and spirit while caring for patients and their families. Critical Care Nurse, 31(6), 69–70. 10.4037/ccn2011549 22135335

[nop21009-bib-0074] Mitchell, M. , Kean, S. , Rattray, J. , Hull, A. , Davis, C. , Murfield, J. , & Aitken, L. (2017). A family intervention to reduce delirium in hospitalised ICU patients: A feasibility randomised controlled trial. Intensive and Critical Care Nursing, 40, 77–84. 10.1016/j.iccn.2017.01.001 28254205

[nop21009-bib-0075] Morrow, J. , Hunt, S. , Rogan, V. , Cowie, K. , Kopacz, J. , Keeler, C. , Billick, M. , & Kroh, M. (2013). Reducing waste in the critical care setting. Nursing Leadership, 26, 17–26. 10.12927/cjnl.2013.23362 24860948

[nop21009-bib-0078] Mulhall, A. , Le May, A. , & Alexander, C. (1999). Bridging the research‐practice gap: A reflective account of research work. NT Research, 4(2), 119–130. 10.1177/136140969900400206

[nop21009-bib-0080] NascimentoI, K. , & Erdmann, A. (2009). Understanding the dimensions of intensive care: Transpersonal caring and complexity theories. Revista Latino‐Americana De Enfermagem, 17(2), 215–221. 19551275 10.1590/s0104-11692009000200012

[nop21009-bib-0081] Nordgren, L. , & Olsson, H. (2004). Palliative care in a coronary care unit: A qualitative study of physicians' and nurses' perceptions. Journal of Clinical Nursing, 13(2), 185–193. 10.1111/j.1365-2702.2004.00816.x 14723670

[nop21009-bib-0082] Ntantana, A. , Matamis, D. , Savvidou, S. , Giannakou, M. , Gouva, M. , Nakos, G. , & Koulouras, V. (2017). Burnout and job satisfaction of intensive care personnel and the relationship with personality and religious traits: An observational, multicenter, cross‐sectional study. Intensive and Critical Care Nursing, 41, 11–17. 28408074 10.1016/j.iccn.2017.02.009

[nop21009-bib-0083] Nyholm, L. , & Koskinen, C. (2017). Understanding and safeguarding patient dignity in intensive care. Nursing Ethics, 24(4), 408–418. 10.1177/0969733015605669 26419439

[nop21009-bib-0084] O’Connor, D. (2016). The lived experiences of nurses caring for patients at the end of life in clinical settings (Unpublished doctoral thesis). Barry University.

[nop21009-bib-0085] Ogden, R. (2008). Bias. In M. Given (Ed.), The SAGE encyclopedia of qualitative research methods, (1, pp. 60–61). SAGE Publications.

[nop21009-bib-0086] Ogle, K. , & Glass, N. (2014). Nurses' experiences of managing and management in a critical care unit. Global Qualitative Nursing Research, 1, 233339361453261. 10.1177/2333393614532617 PMC5342859 28462287

[nop21009-bib-0087] Piquette, D. , Reeves, S. , & Leblanc, V. (2009). Interprofessional intensive care unit team interactions and medical crises: A qualitative study. Journal of Interprofessional Care, 23(3), 273–285. 10.1080/13561820802697818 19387907

[nop21009-bib-0088] Polit, D. , & Beck, C. (2008). Nursing research: Generating and assessing evidence for nursing practice (8th ed). Wolters Kluwer Health, Lippincott Williams & Wilkins.

[nop21009-bib-0089] Polit, D. , & Beck, C. (2017). Nursing research: Generating and assessing evidence for nursing practice (10th ed). Wolters Kluwer Health, Lippincott Williams & Wilkins.

[nop21009-bib-0090] Ranse, K. , Yates, P. , & Coyer, F. (2012). End‐of‐life care in the intensive care setting: A descriptive exploratory qualitative study of nurses’ beliefs and practices. Australian Critical Care, 25(1), 4–12. 10.1016/j.aucc.2011.04.004 21565520

[nop21009-bib-0091] Reiss, P. (2005). Patient perceptions of quality of nursing care as evidenced by nurse caring behaviors. Chicago: Loyola University Chicago.

[nop21009-bib-0092] Richardson, A. , Turnock, C. , Harris, L. , Finley, A. , & Carson, S. (2007). A study examining the impact of 12‐hour shifts on critical care staff. Journal of Nursing Management, 15(8), 838–846. 10.1111/j.1365-2934.2007.00767.x 17944610

[nop21009-bib-0093] Roberts, D. (2007). Ethnography and staying in your own nest. Nurse Researcher, 14(3), 15–24. 10.7748/nr2007.04.14.3.15.c6029 17494465

[nop21009-bib-0094] Roberts, T. (2009). Understanding ethnography. British Journal of Midwifery, 17(5), 291–294.

[nop21009-bib-0095] Robstad, N. , Söderhamn, U. , & Fegran, L. (2018). Intensive care nurses’ experiences of caring for obese intensive care patients: A hermeneutic study. Journal of Clinical Nursing, 27(1–2), 386–395. 10.1111/jocn.13937 28639344

[nop21009-bib-0096] Rose, L. (2011). Interprofessional collaboration in the ICU: How to define? Nursing in Critical Care, 16(1), 5–10. 10.1111/j.1478-5153.2010.00398.x 21199549

[nop21009-bib-0097] Royal College of Nursing (2005). Managing your stress: A guide for nurses. Retrieved from http://www.rcn.org.uk/__data/as‐sets/pdf_file/0008/78515/001484.pdf

[nop21009-bib-0098] Rushton, C. (2006). Defining and addressing moral distress tools for critical care nursing leaders. AACN Advanced Critical Care, 17(2), 161–168. 10.1097/00044067-200604000-00011 16767017

[nop21009-bib-0099] Rushton, C. , Reina, M. , & Reina, D. (2007). Building trustworthy relationships with critically ill patients and families. AACN Advanced Critical Care, 18(1), 19–30. 10.4037/15597768-2007-1004 17284945

[nop21009-bib-0100] Saumure, K. , & Given, L. (2008). Rigor in qualitative research. In M. Given (Ed.), The SAGE encyclopedia of qualitative research methods (2, pp. 795–796). SAGE Publications.

[nop21009-bib-0101] Schmalenberg, C. , & Kramer, M. (2007). Types of intensive care units with the healthiest, most productive work environments. American Journal of Critical Care, 16(5), 458–468. 10.4037/ajcc2007.16.5.458 17724243

[nop21009-bib-0102] Scott‐Findlay, S. , & Estabrooks, C. A. (2006). Mapping the organizational culture research in nursing: A literature review. Journal of Advanced Nursing, 56(5), 498–513. 10.1111/j.1365-2648.2006.04044.x 17078826

[nop21009-bib-0103] Shorideh, F. , Ashktorab, T. , & Yaghmaei, F. (2012). Iranian intensive care unit nurses’ moral distress: A content analysis. Nursing Ethics, 19(4), 464–478. 10.1177/0969733012437988 22691602

[nop21009-bib-0104] Siffleet, J. , Williams, A. , Rapley, P. , & Slatyer, S. (2015). Delivering best care and maintaining emotional well‐being in the intensive care unit: The perspective of experienced nurses. Applied Nursing Research, 28(4), 305–310. 10.1016/j.apnr.2015.02.008 26608430

[nop21009-bib-0105] Smith‐Sullivan, K. (2008). Diaries and journals. In M. Given (Ed.), The SAGE encyclopedia qualitative research methods (1, pp. 213–215). SAGE Publications.

[nop21009-bib-0106] Speziale, H. (2007). Designing data generation and management strategies. In H. J. Speziale , & C. Carpenter (Eds.), Qualitative research in nursing: Advancing the humanistic imperative (4th ed., pp. 35–58). Lippincott Williams & Wilkins.

[nop21009-bib-0107] Spradley, J. P. (1980). Participant observation. Holt, Rinehart and Winston.

[nop21009-bib-0108] Taylor, C. , Lillis, C. , LeMone, P. , & Lynn, P. (2008). Fundamentals of nursing: The art and science of nursing care (6th ed). Lippincott Williams & Wilkins.

[nop21009-bib-0109] Templeman, J. (2015). An ethnographic study of critical care nurses' experiences following the decision to withdraw life‐sustaining treatment from patients in a UK intensive care unit. University of Salford. (Doctoral thesis).

[nop21009-bib-0110] Tirgari, B. , Khandani, B. , & Forouzi, M. (2013). Spiritual care: Iranian critical care nurses' perception. Asian Journal of Nursing Education and Research, 3(4), 262–266.

[nop21009-bib-0111] Todaro‐Franceschi, V. (2013). Critical care nurses' perceptions of preparedness and ability to care for the dying and their professional quality of life. Dimensions of Critical Care Nursing, 32(4), 184–190. 10.1097/DCC.0b013e31829980af 23759913

[nop21009-bib-0112] Tracy, M. , & Ceronsky, C. (2001). Creating a collaborative environment to care for complex patients and families. AACN Advanced Critical Care, 12(3), 383–400. 10.1097/00044067-200108000-00007 11759357

[nop21009-bib-0113] Tremper, R. (2004). Mediastinal exploration in the surgical intensive care unit. Dimensions of Critical Care Nursing, 23(1), 24–30. 10.1097/00003465-200401000-00007 14734897

[nop21009-bib-0114] Tyler, H. (2017). Nursing education on caring for the dying (Doctoral thesis). Walden University Minnesota.

[nop21009-bib-0115] Virginio, B. , Escudeiro, C. , Christovam, B. , Silvino, Z. , Guimarães, T. , & Oroski, G. (2014). Death and organ donation from the point of view of nurses: A descriptive study. Online Brazilian Journal of Nursing, 13(1), 92–101. 10.5935/1676-4285.201404164

[nop21009-bib-0116] Walker, W. , & Deacon, K. (2016). Nurses' experiences of caring for the suddenly bereaved in adult acute and critical care settings, and the provision of person‐centered care: A qualitative study. Intensive and Critical Care Nursing, 33, 39–47. 10.1016/j.iccn.2015.12.005 26853502

[nop21009-bib-0117] Welch, K. , & Barksby, J. (2011). Supporting a client in intensive care. Learning Disability Practice, 14(8), 14–17. 10.7748/ldp2011.10.14.8.14.c8739

[nop21009-bib-0118] Wikström, A.‐C. , Cederborg, A.‐C. , & Johanson, M. (2007). The meaning of technology in an intensive care unit—An interview study. Intensive and Critical Care Nursing, 23(4), 187–195. 10.1016/j.iccn.2007.03.003 17467992

[nop21009-bib-0119] Winters, J. (2012). The experience of newly licensed registered nurses in caring for older adults during acute hospitalization. The University of California.

